# Regulation of common neurological disorders by gut microbial metabolites

**DOI:** 10.1038/s12276-021-00703-x

**Published:** 2021-12-02

**Authors:** Jeongho Park, Chang H. Kim

**Affiliations:** 1 College of Veterinary Medicine and Institute of Veterinary Science, Kangwon National University, Chuncheon, Gangwon 24341 Republic of Korea; 2 Department of Pathology, University of Michigan School of Medicine, Ann Arbor, MI 48109 USA; 3 Mary H. Weiser Food Allergy Center, Center for Gastrointestinal Research, and Rogel Center for Cancer Research, University of Michigan School of Medicine, Ann Arbor, MI 48109 USA

**Keywords:** Autoimmune diseases, Chronic inflammation

## Abstract

The gut is connected to the CNS by immunological mediators, lymphocytes, neurotransmitters, microbes and microbial metabolites. A mounting body of evidence indicates that the microbiome exerts significant effects on immune cells and CNS cells. These effects frequently result in the suppression or exacerbation of inflammatory responses, the latter of which can lead to severe tissue damage, altered synapse formation and disrupted maintenance of the CNS. Herein, we review recent progress in research on the microbial regulation of CNS diseases with a focus on major gut microbial metabolites, such as short-chain fatty acids, tryptophan metabolites, and secondary bile acids. Pathological changes in the CNS are associated with dysbiosis and altered levels of microbial metabolites, which can further exacerbate various neurological disorders. The cellular and molecular mechanisms by which these gut microbial metabolites regulate inflammatory diseases in the CNS are discussed. We highlight the similarities and differences in the impact on four major CNS diseases, i.e., multiple sclerosis, Parkinson’s disease, Alzheimer’s disease, and autism spectrum disorder, to identify common cellular and molecular networks governing the regulation of cellular constituents and pathogenesis in the CNS by microbial metabolites.

## Neurological disorders: bugs that benefit the brain

Disturbances of the microbial ecosystem in our gut create physiological disturbances that can accelerate the onset of neurological disease. The intestinal microbiome generates a diverse array of metabolites that can boost metabolic health, immune function, and other essential biological processes in the human host. Jeongho Park of Kangwon National University, Chuncheon, South Korea, and Chang Kim at the University of Michigan, Ann Arbor, USA, have reviewed research into how disruption of the healthy microbiome may contribute to Alzheimer’s, Parkinson’s, multiple sclerosis, and autism spectrum disorders. These studies indicate that such a “dysbiotic” state is generally associated with increased levels of inflammation. These disturbances are not sufficient to trigger neurological disorders on their own, but the resulting inflammatory state can act in concert with other biological risk factors to accelerate disease pathology.

## Introduction

The central nervous system (CNS) is known as an immune-privileged tissue system but remains susceptible to inflammatory responses. While well protected by the blood–brain barrier (BBB), nevertheless the CNS hosts many types of inflammatory responses. A mounting body of evidence indicates the presence of close interactions between the neural system and gut microbes. These interactions involve endocrine, neurological, metabolic and immunological communication. The CNS and enteric nervous system (ENS) communicate via neurotransmitters, hormones, and metabolites^1,2^. Gut microbial metabolites are transported to the CNS, wherein they regulate CNS cells and immune cells. This review will examine our current understanding of how gut microbial metabolites impact four major CNS diseases and highlight the common disease-modifying functions of major microbial metabolites.

Multiple sclerosis (MS) is the most common form of inflammatory disease in the CNS and is associated with autoimmune-mediated demyelination^3,4^. Other less frequent inflammatory diseases include Rasmussen’s encephalitis^5^ and neuromyelitis optica (NMO)^6^. These diseases involve immune cell infiltrates in the brain and spinal cord, resulting in neuritis and myelitis^7,8^. Antigen-specific T and B lymphocytes are frequent immune cell infiltrates in CNS lesions. Inflammatory responses in the CNS are induced or suppressed by different types of T lymphocytes, such as Th1 and Th17 cells and Tregs^9,10^. Antibodies and B cells can augment or alter the pathogenesis of CNS inflammation^11^. Other cell types, such as microglia, macrophages and astrocytes, actively participate in immune cell regulation and neuroinflammation^12^. An increasing body of evidence indicates that other neurological diseases, such as Parkinson’s disease (PD), Alzheimer’s disease, and autism spectrum disorder (ASD), also involve significant inflammatory responses^13–15^. Therefore, we focus this review on these four CNS diseases, which have distinct tissue involvement, inflammatory responses and pathogenesis.

In recent years, we have witnessed a plethora of research on the functions of gut microbiota and their metabolites in regulating inflammatory responses in the CNS^16–19^. The intestine is rich in dietary materials and host secretions such as bile acids and mucins, which are subsequently metabolized by the cooperative activities of host enzymes and then by the gut microbiome. Various dietary fibers and complex carbohydrates can reach the terminal ileum and colon, which host the majority of the gut microbiome in the body. Microbial fermentation of sugars derived from carbohydrates in the colon produces short-chain fatty acids (SCFAs) such as acetate (C2), propionate (C3) and butyrate (C4)^20^. Another major group of metabolites includes tryptophan (Trp) metabolites. Dietary tryptophan is converted into indole derivatives, including indole-3-acetic acid (IAA), indole-3-propionic acid (IPA), indole-3-aldehyde (IAld), indole-3-acetaldehyde (IAAld), and indoleacrylic acid. Serotonin (5-HT), a neurotransmitter, is also produced in the gut lumen from Trp^21–24^. Primary bile acids are produced from cholesterol in the liver and are dehydroxylated by gut microbes to generate secondary bile acids^25^. These gut microbial metabolites function as agonists for various host receptors and intracellular molecules, such as G-protein-coupled receptors (GPCRs) and transcription factors, including GPR43, GPR41, TGR5, aryl hydrocarbon receptor (AhR), pregnane X receptor (PXR), farnesoid X receptor (FXR), vitamin D receptor (VDR), and others^26^. Moreover, many of these metabolites, particularly SCFAs, are metabolized in host cells to generate energy. Microbial metabolites regulate numerous host enzymes, such as histone deacetylases (HDACs). In general, microbial metabolites regulate host gene expression and metabolic activity in both immune cells and tissue cells. Importantly, these metabolites directly and indirectly affect the CNS to exert their regulatory functions^19,27,28^.

## Basic functions of gut microbial metabolites

The most abundant metabolites in the colon are SCFAs, which have a combined luminal concentration of greater than 0.1 M in the human colon^29^. The three major SCFAs, C2, C3 and C4, function through four G-protein-coupled receptors (GPCRs), GPR41, GPR43, GPR109A and Olfr78^30–32^. In addition, SCFAs are natural HDAC inhibitors, and therefore, they can increase the acetylation and phosphorylation of host proteins such as histones, leading to increased gene expression and cell signaling^33,34^. SCFAs directly inhibit Type I and II HDACs and can also indirectly affect certain Type III HDACs, such as Sirtuin 1^35^. Moreover, SCFAs are metabolized in host cells for energy production and thus have significant regulatory effects on host cell metabolism, leading to increased ATP levels, mTOR activity and fatty acid synthesis^36^. SCFAs are known to boost the differentiation of Th1 and Th17 cells and Tregs from naïve CD4 T cells, depending on the immunological milieu^36^. SCFAs increase IL-10 expression by lymphocytes (T and B cells) and macrophages^37,38^ in a manner largely mediated by their HDAC inhibitory activity. SCFAs regulate the activity of innate lymphocytes; they increase the number and activity of Group 3 innate lymphoid cells (ILC3s) but suppress Group 2 innate lymphoid cells (ILC2s) through GPCR activation and HDAC inhibition, respectively^39–41^. SCFAs also induce tolerogenic macrophages and dendritic cells by both GPCR activation and HDAC inhibition^42^.

Certain amino acid metabolites produced by microbes are also important regulators of host cells. Phenylalanine and tyrosine are precursors of dopamine, a key neurotransmitter, in the host, and some commensal bacteria produce amines related to these amino acids that activate host GPR56^43^. Trp is metabolized by both host cells and certain gut bacteria to serotonin (5-hydroxytryptamine), kynurenine (Kyn) and indole derivatives, which function as neurotransmitters and metabolic regulators^24^. Certain indole derivatives, such as IAA, IPA, and IAld, function as ligands for AhR, a member of the basic helix-loop-helix transcription factor family. These metabolites can also regulate host cells through EIF5a, IR76B, and PXR^44^. Certain microbial species, such as *Peptostreptococcus russellii*^45^ and Lactobacillus species^46,47^, can effectively produce indole derivatives^45–47^. Dietary indole derivatives, such as indole-3-carbinol (I3C) and diindolylmethane (DIM), can increase the activity of Tregs but suppress the generation of myelin oligodendrocyte glycoprotein (MOG)-specific Th17 cells^48^. Polyamines such as putrescine and spermidine are produced by intestinal bacteria and are present at 0.5 to 1 mM in the human colon^49^. Polyamines support the hypusine modification of eukaryotic translation initiation factor 5A (eIF5A) and promote polypeptide translation and cell proliferation^50^. Polyamines also have suppressive effects on Th17 cells^51^. Similarly, phytochemical metabolites, such as ginsenosides, resveratrol, and I3C, activate AhR to exert some of their regulatory functions^52,53^. Ginsenosides suppress Th1 and Th17 cells but increase Treg activity^54^. Resveratrol suppresses Th17 activity^55^.

Primary bile acid metabolites, such as cholic acid and chenodeoxycholic acid (CDCA), are made from cholesterol in the liver. Secondary bile acid metabolites, such as deoxycholate (DCA) and lithocholate (LCA), are formed by bacterial 7α-dehydroxylation of primary bile acids. Secondary bile acids activate multiple host receptors, such as PXR, VDR, LXR, FXR, TGR5, retinoid-related orphan receptor-γt (RORγt), and FoxP3^25,56^. 3-OxoLCA inhibits Th17 polarization by binding to RORγt, whereas isoallo-LCA enhances Treg generation, in part by producing mitochondrial reactive oxygen species that induce FoxP3 expression^57^. Bile acid metabolites have both beneficial and pathogenic functions^58–61^. Primary and secondary bile acids activate the Nod-like receptor protein 3 (NLRP3) inflammasome and IL-1β production in macrophages^61^, which can increase innate immunity but cause chronic inflammation. However, activation of the bile acid receptors FXR and TGR5 is often associated with decreased inflammatory responses^61,62^.

Some microbial metabolites are pathogenic, causing inflammatory diseases and cancer. Pathogenic metabolites include trimethylamine N-oxide (TMAO)^63^, hydrogen sulfide^64^, phenol, p-cresol, N-nitrosamine, ammonia, 4-ethylphenylsulfate^65^, and uric acid^66^. These metabolites are risk factors for MS, AD, and other neurological disorders^67–69^. Certain environmental contaminants, such as the AhR ligand 2,3,7,8-tetrachlorodibenzo-p-dioxin (TCDD), are also pathogenic^70^.

## Regulation of experimental neuroinflammation by microbial metabolites

The regulatory effects of microbiota on experimental neuroinflammation have been reported^71–74^. Rodent experimental autoimmune encephalitis (EAE) models are most frequently used^75^ because they largely mimic human MS. Antigen-induced EAE can be induced by immunization with several neuronal cell antigens, such as MOG, myelin basic protein (MBP), and proteolipid protein (PLP), along with potent adjuvants, such as complete Freund’s adjuvant and pertussis toxin^75^. Demyelination similar to that in MS is also induced by chemicals such as cuprizone (a copper-chelating agent) and lysolecithin^76–78^. Infection with Theiler’s murine encephalomyelitis virus induces demyelinating encephalitis^79,80^. Moreover, the engineered expression of T and B cell receptors specific for CNS antigens causes spontaneous EAE responses in mice^81,82^.

While dysbiosis exacerbates EAE responses^83,84^, studies with germ-free (GF) animals or broad-spectrum antibiotics revealed that the microbiota is required for optimal EAE pathogenesis^85,86^, perhaps because of the general adjuvant effect of the microbiota in inducing immune responses. When specific microbial species or their products are suppressed, EAE activity may either increase or decrease, depending on the function of the affected microbial species^87,88^. For example, segmented filamentous bacteria (SFB), *S. aureus* and *P. heparinolytica* promote the generation of Th17 cells and exacerbate EAE responses^89–91^. Elimination of these Th17-inducing microbial species ameliorated EAE inflammation^85,86^. In contrast, treatment with SCFA-producing bacteria such as *Clostridium tyrobutyricum* ameliorated EAE immune responses^92^. Thus, the impact of the microbiota on CNS inflammation depends on the function of the altered microbial species in the gut^84^.

It is thought that the microbiota affects neuroinflammation by producing immune regulatory products and metabolites. For example, polysaccharide A (PSA), a capsular polysaccharide produced by *Bacteroidetes fragilis*, has significant immune regulatory function^93^. Oral administration of PSA suppressed demyelination and delayed the onset of EAE activity^94^. In this previous study, PSA increased the number of CD103^+^ dendritic cells (DCs) in draining lymph nodes and induced IL-10-producing T cells. PSA promoted Toll-like receptor 2 (TLR2) activation, which induced CD39^+^ regulatory T cells, in part by modulating DCs. In addition, poly-γ-glutamic acid (γ-PGA) has suppressive effects on EAE activity^95^. This finding is in line with decreased Th17 cell but increased Treg activity in the CNS following the administration of γ-PGA, which activates TLR4-MyD88-dependent and TLR4-MyD88-independent pathways to regulate T cell activity^95^. In contrast, some bacterial products can exacerbate CNS inflammation. Lipopolysaccharide (LPS) treatment accelerated inflammation-mediated demyelination in rats^96^. It is well established that peptidoglycans, the major cell wall component of gram-positive bacteria, promote DC maturation through TLR2 activation, which increases the secretion of proinflammatory cytokines and promotes effector T cell generation during EAE development^89^. Thus, microbial TLR ligands can have both inflammatory and suppressive roles in neuroinflammation.

**Fig. 1:**
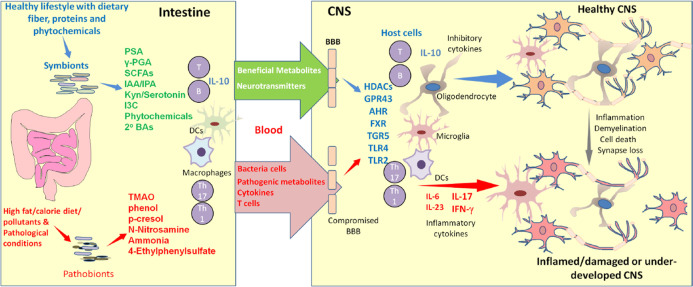
Microbial metabolites regulate CNS development, integrity, and inflammation. Microbial metabolites positively and negatively influence CNS development and inflammatory responses. In the best case, beneficial metabolites are produced in symbiosis with a balanced population of diverse microbes in the gut. Together, these microbes produce myriad metabolites that are beneficial for the host. In dysbiosis, the production of harmful metabolites is increased while that of beneficial metabolites is decreased. In general, beneficial metabolites, such as SCFAs and Trp metabolites, reinforce the integrity of the gut barrier and BBB and support the functional maturation of CNS cells such as microglia, oligodendrocytes and astrocytes. Thus, these metabolites support CNS formation, neurological function, and the development of regulatory immune cells for immune tolerance. Moreover, these metabolites suppress harmful immune responses, such as the generation of pathogenic Th17 cells. These functions are mediated in part by various host receptors, such as GPCRs, transcription factors, nuclear ligand receptors (PXR and FXR), and TLRs. Conversely, harmful metabolites weaken the gut barrier and BBB and cause systemic inflammatory responses, neuronal cell death and tissue injury (e.g., demyelination), leading to inflammatory conditions that exacerbate CNS diseases. Not only harmful microbial metabolites but also pathogenic bacterial cells and T cells travel from the gut to the CNS to increase inflammation under pathological conditions.

BBB disruption is observed in EAE and MS patients, and this can cause harmful exposure of CNS tissues to microbial products and microbial invasion following gut dysbiosis, leading to amplified inflammatory responses^97,98^. Under normal conditions, the microbiota and SCFAs support the integrity of the BBB^99^; it is important to protect the CNS from the harmful effects of inflammatory responses and toxicants. The BBB integrity is weakened in GF mice, and this defect is normalized by SCFA treatment in drinking water^99^ (Fig. 1). SCFAs, such as C2, C3, or C4, administered in drinking water ameliorated EAE activity. Interestingly, SCFAs appear to support CNS development beyond their immunoregulatory roles^100,101^. Microglial cells in GF mice had defective microglia with altered cell proportions and an immature phenotype, which were corrected by SCFA feeding in a GPR43-dependent manner^100^. In addition, C4 promoted oligodendrocyte maturation in the brain^101^. Thus, SCFAs support the functional maturation of CNS cells (Fig. 1). Increased levels of secondary bile acids, such as DCA, can weaken BBB function, perhaps because these compounds are amphiphilic steroids that loosen the cell membrane^102,103^. Thus, BBB function and associated CNS pathogenesis can be altered by changes in microbial metabolites.

Multiple groups have reported that SCFAs have EAE-suppressing activities (Table 1). Mice with elevated levels of SCFAs after feeding with SCFA water or dietary fiber had increased numbers of regulatory T cells in the intestine and CNS upon MOG immunization^104,105^. In addition, when mice were fed C4, cuprizone-mediated demyelination was ameliorated.^18,74,101,105^. The protective effect of C4 was further validated in a lysolecithin-induced demyelination model^101^. In addition to the major SCFAs (i.e., C2, C3, and C4), valerate (C5) can also regulate EAE activity^27^. C5 increased both regulatory B cells (Bregs) and T cells (Tregs). C5-treated B cells showed increased expression of IL-10, and adoptive transfer of these B cells ameliorated EAE activity. The protective effect was accompanied by decreased Th17 cell activity in the small intestine. C5, well known for its potent HDAC inhibitory activity, increased mTOR activation and glycolysis in lymphocytes^27^. Thus, both epigenetic (HDAC inhibition) and metabolic (mTOR) regulation appears to be involved in the protective effects of SCFAs in EAE pathogenesis.

**Table 1:** Regulation of CNS diseases in animal models by gut microbial metabolites.

Diseases in animal models	Metabolites	Effects of metabolites on disease	Effects of metabolites on cells and molecules	Ref
EAE and related demyelinating diseases	SCFAs	Exacerbation	GPCR-mediated immune cell activation; Increased Th17 polarization	^18^
		Suppression	IL-10 production; Induction of regulatory T and B cells; Decreased MAPK activation; Th1 suppression; HDAC inhibition; Increased glycolysis and AKT/mTOR; Oligodendrocyte maturation	^18,27,102,104,105^
	LCFA	Exacerbation	MAPK activation; Increased Th17 and Th1 activity; Decreased Treg activity	^104,216^
	Trp metabolites (3.4-DAA I3S, I3C, DIM, IPA, IAld)	Suppression	STAT1-mediated suppression of antigen presenting cells; Activation of microglial AhR; Decreased NF-κB activity; Th17 suppression; Treg expansion; Increased SOCS2 activity	^116,150,217^
	PSA	Suppression	Increased activity of CD103^+^ DCs; Induction of IL-10^+^ T cells; TLR2-dependent increase in CD39^+^CD4^+^ T cell activity	^94,217^
	2^ND^ BA (TUDCA)	Suppression	Suppression of inflammatory responses in astrocytes and microglia cells in a GPBAR-dependent manner	^143^
PD models	SCFAs	Exacerbation	Microglia activation; Increased αSyn-mediated motor dysfunction; Activation of microglial and astrocytes; Higher expression of TLR4, Increased activity of TBK1, NF-κB, and TNF-α.	^71,218^
		Suppression	GPR41 activation; Suppression of dopaminergic neuronal loss; Enrichment of C4-producing bacteria; Increased gut occludin expression	^163,164^
	2^ND^ BA (UDCA, and TUDCA)	Suppression	Increased intracellular ATP levels; Enhanced contrast response function; Suppressed JNK activity; Suppressed ROS production	^179,183^
AD models	SCFAs	Exacerbation	Microglial activation; Increased amyloid β plaque deposition via apoE-TREM2	^199^
		Suppression	Increased neuronal activity with hippocampal c-Fos expression; Decreased polymerization of amyloid β; Suppression of NF-κB and COX-2 in microglia	^73,188,195^
ASD models	SCFAs	Exacerbation	Astrocyte activation; Increased TNF-α production; Altered hippocampus structure	^213^
		Suppression	Increased excitatory/inhibitory balance in the prefrontal cortex	^215^

*AhR* aryl hydrocarbon receptor, *BDNF* brain-derived neurotrophic factor, *COX-2* cyclooxygenase-2, *CREB* cyclic AMP response element binding protein, *GPBAR* G-protein-coupled bile acid receptor, *GPCR* G-protein-coupled receptor, *DAA* digestible amino acid, *DI* diindolylmethane, *DIM* 3,3’-diindolylmethane, *HDAC* histone deacetylases, *IAld* indole aldehyde, *IPA* indole-3-propionic acid, *I3C* indole-3-carbinol, *I3S* 3-indoxyl sulfate, *JNK* c-Jun N-terminal kinase, *MAPK* mitogen-activated protein kinase, *mTOR* mechanistic target of rapamycin, *NF-κB* nuclear factor kappa-light-chain-enhancer of activated B cells, *PSA* polysaccharide A, *ROS* reactive oxygen species, *SOCS* suppressor of cytokine signaling, *STAT* signal transducer and activator of transcription, *TBK1* TRIF-TANK binding kinase, *TREM-2* triggering receptor expressed on myeloid cells-2, *Trp* tryptophan, *TUDCA* tauroursodeoxycholic acid, *UDCA* ursodeoxycholic acid.

The effects of SCFA-producing fiber-rich diets on EAE are mixed, and this appears to be due to considerable variation in the dietary fiber composition of high- and low-fiber diets used by different groups. Some groups used a diet containing no fiber (soluble or insoluble) as the control diet^106–108^. In general, soluble fibers such as pectin and inulin produce high levels of SCFAs, whereas insoluble fibers such as cellulose are not efficient sources of SCFAs. Other groups, including ours, compared the effects of high- and low-fiber diets containing the same amount of cellulose but different levels (0, 5, and 20%) of soluble fiber. When a high-fiber diet was compared with a zero-fiber diet, the protective effect was significant^105^. However, when the animal group fed a diet high in soluble fiber (pectin and inulin) was compared with that fed a diet containing zero soluble dietary fiber but the same level of cellulose, the difference was not significant^18^. This finding suggests the involvement of mechanisms other than SCFA production in the beneficial effects of dietary fiber. Despite its low bioavailability and SCFA-producing ability, cellulose seems to have a suppressive effect on EAE activity^84^. Cellulose decreased the number of neutrophils and Th2 cells in a spontaneous opticospinal encephalomyelitis mouse model expressing MOG-specific T and B cell antigen receptors^84^. While cellulose did not significantly change SCFA levels, it changed the microbiome composition in the gut, indicating that the protective effect of certain dietary fibers, such as cellulose, appears to be more dependent on microbial changes than on SCFA production^108^.

SCFAs appear to regulate EAE responses via several cellular and molecular mechanisms. SCFAs may function through astrocytes and microglial cells, which produce both suppressive and inflammatory cytokines and influence EAE responses. SCFAs can also act through immune cells such as Th17 cells and regulatory T cells, which differentially regulate inflammatory responses. Several cell types can produce IL-10 in response to SCFAs. Major cell types that produce IL-10 are T cells and macrophages, and this production can be further increased by SCFAs^38,109–111^ (Fig. 1). In our own study, SCFAs increased IL-10 production by microglial cells, and IL-10 production was required for the protective effect of SCFAs on EAE activity^18^.

While SCFAs have suppressive effects on EAE activity, their G-protein-coupled receptors, such as GPR43, can function unexpectedly to exacerbate EAE activity^18^. The mechanism is not entirely clear at this point, but the data imply that the protective function of SCFAs is perhaps mediated mainly through non-GPCR-dependent mechanisms. In this regard, the HDAC inhibitory activity of SCFAs may be important. GPCR activation by SCFAs can increase immune activity, including invigorating epithelial responses and activating inflammasomes in the gut^112,113^. We speculate that GPCR activation by SCFAs in the CNS could promote similar inflammatory responses. In this regard, the function of SCFAs in regulating EAE activity appears to be complex. When transferred into host mice, SCFA-treated MOG-specific effector Th17 cells have greater EAE activity than similarly prepared control Th17 cells^18^, indicating that SCFAs can affect both the inflammatory and regulatory arms of the CNS immune system. More work is required to understand the effects of SCFAs on key immune and CNS cell types, such as T and B cells, ILCs, glial cells, neurons, macrophages, and dendritic cells.

Indole derivatives and certain environmental contaminants, such as the AhR ligand 2,3,7,8-tetrachlorodibenzo-p-dioxin (TCDD), have been studied for their regulatory effects on EAE activity. AhR activation by FICZ and 2-(1′H-indole-3′-carbonyl)-thiazole-4-carboxylic acid methyl ester (ITE) (Trp-derived indole metabolites) suppressed EAE responses^114,115^, whereas AhR activation by TCDD exacerbated EAE pathogenesis^70^. Similarly, FICZ and TCDD differentially affected Th17 cells, Tregs, DCs, and astrocytes. The administration of dietary indoles was effective at suppressing EAE development^48^. Moreover, dietary indoles significantly ameliorated existing EAE^48,116^; this protection is thought to be mediated by the AhR-mediated expansion of regulatory T cells. These Trp-derived metabolites appear to suppress CNS inflammation by decreasing proinflammatory cytokine expression and inflammatory monocyte infiltration^47,48,116^, which may involve activation of the type-I IFN pathway in astrocytes to limit CNS inflammation in an AhR-dependent manner. Similarly, cruciferous plant-derived AhR ligands such as glucosinolates suppressed EAE pathogenesis^117^.

## Significant associations between microbial metabolites and MS in humans

In humans, MS manifests in several ways with different patterns of disease progression and relapse; the common categorizations of MS include clinically isolated syndrome (CIS), relapsing-remitting MS (RRMS), primary progressive MS (PPMS), and secondary progressive MS (SPMS)^118,119^. RRMS, with repeated relapses and remissions, is most common (~85%). Most people with RRMS progress to SPMS with steadily worsening symptoms with or without remissions and relapses. PPMS is rarer (~10%), with slowly worsening symptoms from disease onset without remissions and relapses.

Changes in gut luminal microbial composition have been observed along with the abnormal invasion of microbes in the brains of RRMS patients^120^. RRMS patients have increased levels of *Actinobacteria* but decreased levels of *Firmicutes* and *Bacteroidetes* in feces. Within the *Firmicutes* family, fourteen species within Clostridium clusters XIVa and IV were found to be decreased in MS patients^121^. A cohort study found decreased *Prevotella_9* but increased *Streptococcus* species, along with reduced levels of fecal SCFAs, in MS patients. In general, Tregs and Th17 cells in the blood indicate tolerogenic and inflammatory activity, respectively^122^. A positive correlation between *Streptococcus* species and Tregs was reported, and an inverse correlation between *Prevotella_9* and Th17 cells was observed. Increased numbers of microbial peptidoglycan-containing macrophages and dendritic cells were detected in the brains of MS patients. Thus, MS patients have both dysbiosis and microbial invasion of the brain^120,123^. CCR9 is the small intestine-homing receptor for lymphocytes^124,125^ and is important for thymocyte localization in the thymic medulla^126,127^. Interestingly, CCR9^+^CD4^+^ T cells were found in the cerebrospinal fluid (CSF) of MS patients. T cell migration into inflamed CNS tissues involves another chemokine receptor, CCR6, that is highly expressed by Th17 cells^128^. CCR9^+^CD4^+^ T cells express RORγ and secrete IL-17 and IFN-γ in MS patients. Antibiotic treatment decreased these Th17 cells, suggesting that inflammatory CCR9^+^CD4^+^ T cells are induced by microbes^129^. While further study is required to determine the origin of these T cells, they appear to be generated in the intestine following dysbiosis and neural inflammation. These data imply the active movement of microbes and immune cells from the gut to CNS tissues under inflammatory conditions.

Normally, antigen-induced EAE induction in animals requires the use of potent adjuvants to break immune tolerance to self-antigens^75^. Fecal microbial transfer (FMT) from MS patients to animals induced EAE development following immunization without any adjuvant. FMT decreased certain microbial species, such as those in the *Sutterella* genus, and decreased IL-10 expression by T cells^16,130^. The effect of SCFA-generating dietary fibers on MS activity has been studied. A diet rich in plant fibers increased the fecal presence of the *Lachnospiraceae* phylum in MS patients and increased the numbers of Tregs and IL-10-producing PD-L1^+^ monocytes in the blood. While a fiber-rich diet failed to change MS clinical activity, patients on a Western diet had increased MS clinical activity^131^.

Negative correlations have been found between SCFA levels and T cell activity in MS patients. Higher fecal and blood SCFA levels were associated with increased Treg and suppressed Th1 cell activity^122,131,132^. Compared with healthy individuals, MS patients have decreased levels of blood C4 and SCFA-producing microbial species^118^. In line with the dysbiosis and reduced levels of SCFAs in MS patients^118,133^, decreased levels of all major SCFAs, such as C2, C3, and C4, were found in the blood of SPMS patients compared with that of healthy controls^18^. In addition, gut SCFA levels were decreased in RRMS patients^122^. In this regard, C3 increased Treg activity and decreased episodes of MS relapse^134^. In another study, fecal C3 levels were decreased in both RRMS and SPMS patients^132^. The therapeutic effect of C3 in MS patients was recently reported^134^. Regardless of MS subtype, C3 supplementation alleviated the clinical symptoms of MS, and MS patients with increased levels of SCFA-producing gut bacteria showed limited inflammatory T cell activity. C3 also increased regulatory T cells that produce IL-10, altered the expression of many genes in T cells, and increased oxygen consumption in T cell mitochondria. However, increased levels of plasma C2 and increased numbers of circulating Th17 cells were observed in MS patients with severe disability^135,136^. Because C2 is also produced by host cells under inflammatory and infection conditions^137^, the increased levels of C2 could originate from the host rather than microbial cells. These data, while contradictory to other results, seem to be in line with the effect of C2 on Th17 cell generation^109^. Therefore, more studies are required to establish the possible associations between SCFA levels and MS subtypes. More data may enable the utilization of SCFAs as biomarkers for MS activity and gut microbial status in MS patients.

Oral Trp administration boosted serotonin but decreased plasma cortisol levels in patients with neurological diseases. Acute MS and other neurologic symptoms were alleviated^138,139^. Dietary Trp can be metabolized by gut microbes or host cells after enteric absorption. While gut microbes produce various indole metabolites, host cells produce Kyn, kynurenic acid (KA) and quinolinic acid (QA) via the kynurenine pathway. The levels of urinary Kyn were decreased in RRMS patients^140^. KA levels were increased in RRMS patients but decreased in PPMS patients, while the level of QA was increased in most MS patients^141^. Trp administration improved the cognition and memory function of MS patients^140,142–144^. Pediatric MS is less frequent than adult MS, but relapses are more frequent and possibly more inflammatory in affected pediatric patients^145^. Increased *Actinobacteria* but decreased *Clostridiales* were observed in pediatric MS patients^146^. Decreased levels of Trp and indole lactate were also observed in pediatric MS patients. Interestingly, higher Kyn levels were associated with an increased relapse rate^142^. Many Trp metabolites, such as I3S, tryptamine (TA), IAA, Kyn and KA, function as AhR ligands^147,148^. KA also activates another host receptor, GPR35^149^. Trp metabolites increase AhR expression on microglia and astrocytes and control the inflammatory activities of these cells^116,150^. Secondary bile acids also regulate astrocytes by upregulating GPBAR1. Tauroursodeoxycholic acid (TUDCA), a taurine conjugate of ursodeoxycholic acid (UDCA), suppressed microglial and astrocytic polarization (Table 2)^143^. Overall, multiple gut metabolites can modulate microglial cells and astrocytes to suppress MS pathogenesis (Fig. 1).

**Table 2:** Association of CNS diseases and gut microbial metabolites in humans.

Diseases	Metabolites or their precursors	Effects of metabolites on disease	Effects of metabolites on cells and molecules	Ref
MS	SCFAs	Exacerbation	Induction of IL-17^+^CD8 T cells; Increased C2 levels in MS patient serum	^135,136^
	SCFAs	Suppression	Increased Tregs but decreased Th1 cells; Increased mitochondrial oxidation	^118,122,132,134^
	Trp and Trp metabolites (indole, I3S, I3P, I3A)	Suppression	Limited the pathogenic activity of microglia and astrocytes; AhR activation	^116,150^
	Secondary bile acids	Suppression	Activation of GPBAR1 in astrocytes; TUDCA-mediated suppression of microglial and astrocytic inflammatory polarization	^143^
PD	SCFAs	Exacerbation	Dysbiosis-mediated gut leakage	^219^
		Suppression	Decreased Prevotellaceae and Enterobacteriaceae; Increased blood levels of CXCL8 and IL-1β; Increased gut permeability	^160,167,174^
	Secondary bile acids	Exacerbation	Decreased lipid metabolism; Dysbiosis and elevated BA levels	^165,166^
	TMAO	Exacerbation	Increased α-syn aggregation and inflammation	^220^
AD	SCFAs	Exacerbation	Increased production of inflammatory cytokines; Endothelial dysfunction	^187^
		Suppression	Increased IL-10 levels	^187^
	Tryptophan	Suppression	Decreased tryptophan but increased blood Kyn/Trp ratio in AD patients	^201^
	Secondary Bile acid	Exacerbation	Increased DCA but decreased primary BAs (cholic acid); Increased levels of TCA, 3-DCA and UDCA in the brain of AD patients	^202,203^
ASD	SCFAs	Exacerbation	Increased levels of butyrogenic bacteria; Increased levels of fecal SCFAs in ASD patients	^221,222^
		Suppression	Decreased fecal levels of SCFAs in ASD patients	^211,212^

*AhR* aryl hydrocarbon receptor, *α-syn* α-synuclein, *BA* bile acid, *DCA* deoxycholic acid, *I3A* *GPBAR* G-protein-coupled bile acid receptor, *I3A* indole-3-carboxaldehyde, *I3P* indole-3-propionic acid, *I3S* indoxyl 3-sulfate, *TCA* trichloroacetic acid, *TGF* transforming growth factor, *TMAO* trimethylamine N-oxide, *Trp* tryptophan, *TUDCA* tauroursodeoxycholic acid, *UDCA* ursodeoxycholic acid, *VEGF* vascular endothelial growth factor.

## Microbial metabolites and Parkinson’s disease

Parkinson’s disease (PD) is caused by the loss of dopaminergic neurons in the substantia nigra (SN), leading to dopamine deficiency in the midbrain; in this region, dopamine is important for the regulation of body movement^151^. A number of genes and their polymorphisms have been implicated in PD pathogenesis^152^. The presence of neurotoxic protein inclusions called Lewy bodies, which are composed of α-synuclein oligomers, in the midbrain is a pathological feature of PD^153,154^. Th1 and Th17 cell-driven inflammation, potentially induced by Lewy bodies, plays significant roles in PD pathogenesis^155,156^. Because of the inflammatory nature of PD and the potential roles of microbiota and gut metabolites in regulating PD, we also include a discussion of PD in this review (Tables 1, 2). Both genetics and environmental factors, such as diet and exposure to neurotoxic chemicals, play significant roles in PD pathogenesis. Genetic risk factors include autosomal dominant mutations in the alpha-synuclein (SNCA), leucine-rich repeat kinase 2 (LRRK2) and vacuolar protein sorting ortholog 35 (VPS35) genes as well as autosomal recessive mutations in the PTEN-induced kinase 1 (PINK1), DJ-1 (also known as PARK7) and PARKIN genes^157^. Interestingly, polymorphisms in the LRRK2 gene are commonly found in PD and inflammatory bowel disease patients^158,159^.

Gut dysbiosis has been observed in PD patients^71,160,161^. Gut microbiota can regulate PD pathogenesis in animal models^71,162–164^. Mice colonized with fecal microbiota from PD patients developed PD-like pathological features, including α-synuclein aggregation in the brain^71^. Metabolite profiling of PD patients revealed decreased metabolism of unsaturated fatty acids and increased levels of secondary bile acid metabolites such as deoxycholic acid (DCA) and lithocholic acid (LCA). Importantly, significant decreases in SCFA-producing bacteria and decreased microbial carbohydrate processing activity were detected in a group of PD patients with reduced mobility^165–167^.

Among the LRRK2 gene mutations associated with PD, the G2019S mutation that affects the kinase domain is the most frequent^168^. The immunological characteristics of transgenic rats expressing human LRRK2 G2019S were examined^169^. Defective myelopoiesis and a decreased Th17 cell frequency were observed in these rats when they were challenged with colitis-inducing agents, such as 2,4,6-trinitrobenzene sulfonic acid (TNBS) and dextran sulfate sodium (DSS). Interestingly, we observed increased levels of Bacteroidetes in this animal model^169^. The MitoPark (MP) mouse model was created by deleting the mitochondrial transcription factor A (TFAM) gene in dopaminergic neurons^170^. Decreased gastrointestinal tract motility and gut dysbiosis were observed in this model. Moreover, increased levels of *Prevotella* were observed in this model and in PD patients. Young rats fed permethrin, a pesticide, developed PD-like symptoms and increased gut permeability. These animals also developed dysbiosis and showed decreased SCFA production.

The protective effects of valproic acid (a SCFA-related HDAC inhibitor) and C4 were observed in a model of PD induced with the chemicals manganese and 1-methyl-4-phenyl-1,2,3,6-tetrahydropyridine^160,163,171–174^. Interestingly, a synthetic FFAR3 (GPR41) agonist (AR420626) corrected the movement disorder and neuronal loss in a 6-hydroxydopamine (OHDA, a neurotoxin)-induced PD mouse model^163^. In contrast, the microbiota and SCFAs were reported to promote PD pathogenesis in α-synuclein-transgenic mice^71^. Other researchers have also reported that SCFA administration exacerbates PD pathogenesis in another animal model^175^. The pathogenic role of SCFAs is unexpected but corresponds with the role of these metabolites in promoting the generation of inflammatory Th1 and Th17 cells, as reported previously by our group^109,176^. While these animal models provide insight into PD pathogenesis, the experimental conditions and pathogenesis in these models differ greatly from each other and from PD patients. Thus, the effects of SCFAs on PD pathogenesis are unclear, and more studies are required.

A previous study reported potential changes in bile acid metabolism with increased levels of secondary bile acids, such as LCA and DCA^165^. In a latent PD model in which α-syn fibrils were injected into the olfactory bulb to induce PD-like pathogenesis, the levels of bile acids such as ω-muricholic acid, TUDCA and UDCA were decreased in the blood^177^. Beneficial roles of TUDCA in acute, progressive and nigral transplant animal models of PD have been reported^178–180^. TUDCA ameliorated the decrease in dopaminergic fibers, mitochondrial dysfunction and neuroinflammation in an MPTP-induced animal model^179,181,182^. Moreover, TUDCA suppressed α-synuclein-induced oxidative stress via the activation of Nrf2, JNK and AKT^179,182^. In addition, UDCA decreased mitochondrial dysfunction in skin fibroblasts from patients harboring the LRRK2 G2019S mutation^183^. Thus, secondary bile acids have protective effects against PD pathogenesis.

## Microbial metabolites and Alzheimer’s disease

AD is a progressive neural disease induced by the accumulation of toxic misfolded amyloid β-protein (Aβ) plaques in the brain. In this disease, extracellular β-amyloid plaques form in the basal, temporal, and orbitofrontal neocortex. In severe AD cases, the plaques spread to the hippocampus^184^. Dysbiosis is a risk factor for AD development. Mice humanized with stool from AD patients showed defects in cognitive function^185^. In addition to the gut bacterial composition, the diversity of gut fungal species (mycobiome) in patients with mild cognitive disorder was lower than that in control subjects. Dietary intervention with a Mediterranean ketogenic diet enhanced fungal microbiome diversity and decreased the levels of AD biomarkers in cerebral spinal fluid^186^.

Similar to MS and PD patients, AD patients have decreased levels of SCFAs^186–189^. In particular, AD patients with brain amyloid accumulation and endothelial dysfunction had decreased blood levels of C4^187^. AD pathology is associated with increased blood LPS and inflammatory cytokines. Interestingly, blood C4 and IL-10, but not acetate and valerate, were negatively associated with AD pathology, suggesting a role for SCFAs in AD pathogenesis. Aging and oxidative stress have been suggested to alter the gut microbial community and thus reduce the levels of SCFAs. Caloric restriction and dietary antioxidant supplementation can diminish AD development, in part by modulating microbial composition^190–192^. Dietary fiber and SCFAs have been reported to have beneficial effects on AD pathogenesis^193–195^. SCFA treatment decreased the polymerization of Aβ1-40 or Aβ1-42 peptides into neurotoxic multimeric Aβ forms^188^. Probiotic supplementation increased the hippocampal concentration of SCFAs and reduced anxiety-like behavior in a humanized AD mouse model^73^. The microbiota has been shown to regulate AD pathogenesis in several animal models. For example, amyloidosis with defective long-term synaptic potentiation develops in the APP/PS1 mouse model, created by overexpressing amyloid precursor protein (APP) and a mutant form of presellin 1 (PS1)^196–198^. Fecal microbiota transplantation into APP/PS1 mice increased C4 levels in the colon and increased the expression of synaptic plasticity-related proteins (PSD-95 and synapsin I) in the brain, indicating a decrease in synaptic disorder. The administration of C4-producing bacteria suppressed β-amyloid deposition and effector cytokine release from microglial cells^73,195,199,200^. In AD patients, the blood Kyn/Trp ratio was found to be increased due to decreased Trp levels, indicating an abnormality in Trp metabolism^201^. The levels of secondary bile acids such as DCA, TCA, 3-DCA, and UDCA were increased in AD patients^202,203^. Thus, AD patients have an imbalance in the levels of metabolites that regulate AD pathogenesis. SCFAs have both protective and pathogenic effects, Trp metabolites have protective effects, and bile acid metabolites have overall pathogenic effects on AD (Table 2).

## Impacts on autism spectrum disorder

Autism spectrum disorder (ASD) is a group of developmental disabilities that involve social, behavioral and communication challenges. Both genetic and environmental factors have been identified as risk factors for the development of ASD, which is heritable. For example, chromosome 16p11.2 deletion and 15q11-q13 duplication are risk factors for ASD development. In addition, environmental factors such as maternal age, health status, infection/inflammation, perinatal hypoxia, medication, nutrition, and toxic exposure during fetal or early development are associated with ASD^204,205^. Pathogenic maternal immune activation of the IL-17A pathway was shown to promote ASD development in offspring^206^. The injection of synthetic dsRNA into pregnant dams, mimicking viral infection during pregnancy, induced placental IL-17A expression and fetal brain IL-17 receptor expression in animal models^206^. Th17 inflammation is associated with abnormal cortical development in animal models.

While ASD is different from MS, PD and AD in that it primarily involves CNS developmental issues rather than an ongoing inflammatory disease, a significant body of literature indicates an active role of dysbiosis in ASD. Some ASD patients report GI problems, such as abdominal pain, diarrhea, and food sensitivities^207,208^. Decreased levels of *Firmicutes*, *Fusobacteria* and *Verrucomicrobia* but increased levels of *Bacteroidete*s have been reported in ASD patients^209^. Elevated levels of Th17-inducing gut microbial species, such as segmented filamentous bacteria (SFB), *Bifidobacterium adolescentis*, and certain *E. coli* isolates, during pregnancy were shown to increase ASD development in offspring^210^. Intestinal CD11c^+^ DCs, which recognize microbial TLR3 ligands and produce IL-1β, IL-23, and IL-6, can induce pathogenic Th17 responses^206,210^. The significance of the microbial community in the onset of ASD has been validated in an FMT model^72^. When germ-free mice were humanized with microbes from ASD patient stool, their offspring showed behavioral disorders and decreased locomotion and interactions with other animals. The abundance of *Bacteroides spp*. and *P. merdae* was correlated with increased social behavior. Increased *E. tayi*, however, was associated with decreased social interaction in offspring^72^. Thus, dysbiosis can affect ASD at both the developmental and effector phases.

An altered microbial composition can impact metabolite production. It appears that microbial metabolites can bidirectionally regulate ASD activity. Decreased levels of fecal SCFAs (C2, C3, and C5) were observed in ASD patients^211,212^. Moreover, C4-producing bacteria and fecal SCFA levels were reported to be increased in ASD patients compared with unaffected individuals (Table 2). In a rat ASD model, C3 administration altered the patterning of human neural stem cells, leading to gliosis, disturbed neurocircuitry, inflammatory responses in the hippocampus and increased ASD-like behavior^213,214^. On the other hand, C4 administration improved social behavior and regulated autism-related genes related to the excitatory/inhibitory balance in the prefrontal cortex of mice^215^ (Table 1). Thus, the effect of SCFAs on ASD is currently equivocal. It is interesting that different SCFAs exert different effects on ASD development. Offspring from female animals that received an FMT from human ASD patients had lower levels of 5-aminovaleric acid (5AV) and taurine in the colon; these metabolites are gamma-aminobutyric acid (GABA) receptor agonists. When 5AV and taurine were administered to a mouse model of ASD (i.e., BTBR T+ tf/J mice), ASD-related behavior was suppressed^72^. Thus, microbial metabolites have significant regulatory effects on ASD activity.

## Implications and concluding remarks

The gut is functionally linked to the CNS by immunological mediators, lymphocytes, neurotransmitters, microbes and microbial metabolites. Indeed, it is apparent that most organs and tissues in the body are regulated by gut microbiota and their metabolites, and the CNS is no exception. Importantly, because of the unique (i.e., neurological) functions of the CNS, the impact of gut microbes and their metabolites is manifested quite uniquely in this system. However, the effects of the microbiota and metabolites on different CNS diseases appear to be heterogeneous. Overall, the microbiota and their metabolites influence and strengthen CNS function in the healthy state; for example, SCFAs strengthen the BBB to maintain CNS integrity and protect the CNS from toxic and immunological damage. Various microbial metabolites regulate both inflammatory responses and neuronal activity in the CNS. Gut microbial metabolites promote the maturation of key CNS cells, such as oligodendrocytes, microglial cells and astrocytes, and regulate synapse formation. In addition, the gut microbial metabolites serotonin, dopamine, 5AV and taurine regulate neurotransmission. The production of these metabolites by microbes partly explains the changes in neurological activity following FMT from patients to experimental animals. Certain microbial metabolites, such as SCFAs, secondary bile acids and Trp metabolites, promote immune tolerance by increasing the expression of IL-10 and IL-22 to prevent inflammatory diseases. Under the highly variable pathological conditions of different diseases and hosts, SCFAs and other metabolites induce the generation of effector cells that produce neuroinflammatory cytokines. In this regard, microbial metabolites have important functions in regulating T cells, antigen-presenting cells, microglial cells and astrocytes.

**Fig. 2:**
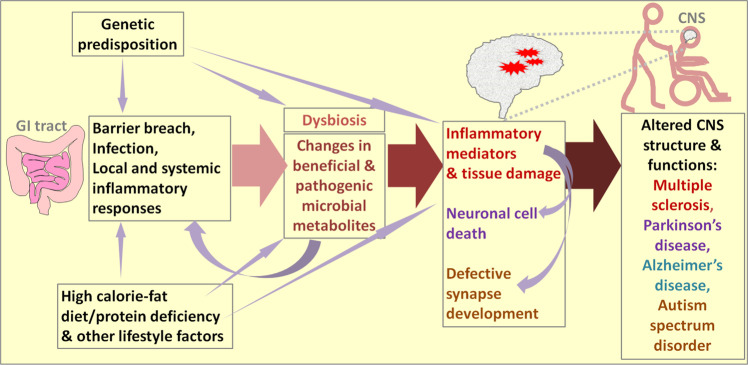
The common regulatory network of microbial metabolites, inflammatory diseases and CNS disorders. The common initiating factors for the four neurological diseases are genetic predisposition and environmental factors, which include diet and lifestyle. Under pathogenic conditions, the intestinal barrier can be breached, and systemic inflammatory responses can occur. These changes can be followed by dysbiosis (i.e., decreased gut microbial diversity leading to decreased levels of beneficial microbes). For example, consumption of a diet high in calories and fat but low in dietary fiber can accelerate pathogenic dysbiosis. As a result, decreased levels of beneficial gut microbial metabolites such as SCFAs, Trp metabolites and phytochemicals are produced, and simultaneously, the production of harmful metabolites such as long-chain fatty acids (LCFAs), certain bile acid metabolites, and toxic microbial metabolites increases, thereby affecting immune and tissue cells in both the intestine and CNS. These changes can decrease immune tolerance, which is important for preventing autoimmune diseases, and exacerbate pathogenic immune responses mediated by inflammatory cells such as Th17 and Th1 cells. These pathogenic inflammatory responses can contribute to tissue damage (demyelination in MS), neuronal cell death (PD and AD), and neuronal synapse development (ASD). Moreover, certain microbial metabolites regulate neurotransmission and, therefore, can directly affect neurological activity.

We discussed the effects of microbiota and their metabolites on the pathogenesis of four different CNS diseases or disorders. A common pattern of regulation emerging from the discussed information is that disease pathogenesis is accelerated under conditions of dysbiosis (Fig. 2). While dysbiosis alone would not cause overt inflammatory responses in the CNS in normal hosts, it appears to increase pathological processes in hosts with underlying disease activity or risk factors. Changes in microbial metabolites are significant modifiers of pathogenesis in the CNS; these changes either positively or negatively affect disease onset and pathogenesis. Moreover, the altered production of microbe-derived neurotransmitters in dysbiosis appears to significantly affect neurological activity.

Despite research progress in this field, significant controversies remain regarding the functions of microbiota and their metabolites in regulating various CNS diseases. We still lack an understanding of the detailed functions of microbial metabolites. Importantly, microbes and microbial metabolites have both pro- and anti-inflammatory roles. What underlies the apparent bidirectional activities of gut microbial metabolites? SCFAs, which are largely anti-inflammatory and disease-suppressing, are sometimes associated with increased CNS disease activity. While more information is needed to elucidate the mechanisms underlying the apparent bidirectional regulation, it is clear that gut microbiota and their metabolites are significant modifiers rather than primary inducers of CNS diseases. Their effects on CNS pathogenesis can be altered depending on host factors, such as the type of inflammatory responses and stage of pathogenesis. Thus, an improved understanding of CNS regulation by microbial metabolites is required. The precise mechanisms by which these metabolites regulate immune responses individually or in combination to suppress or increase pathogenesis in the CNS have yet to be elucidated, particularly for relatively understudied diseases such as PD, AD, and ASD.

## References

[1] Sampson TR, Mazmanian SK (2015). Control of brain development, function, and behavior by the microbiome. Cell Host Microbe..

[2] Silva YP, Bernardi A, Frozza RL (2020). The role of short-chain fatty acids from gut microbiota in gut-brain communication. Front. Endocrinol..

[3] Lassmann H, Brück W, Lucchinetti CF (2007). The immunopathology of multiple sclerosis: an overview. Brain Pathol..

[4] Lucchinetti C (2000). Heterogeneity of multiple sclerosis lesions: implications for the pathogenesis of demyelination. Ann. Neurol..

[5] Bien CG (2002). Destruction of neurons by cytotoxic T cells: a new pathogenic mechanism in Rasmussen’s encephalitis. Ann. Neurol..

[6] Misu T (2013). Presence of six different lesion types suggests diverse mechanisms of tissue injury in neuromyelitis optica. Acta Neuropathol..

[7] Kuhlmann T, Lassmann H, Brück W (2008). Diagnosis of inflammatory demyelination in biopsy specimens: a practical approach. Acta Neuropathol..

[8] Höftberger R, Lassmann H (2017). Inflammatory demyelinating diseases of the central nervous system. Handb. Clin. Neurol..

[9] Bradl M (2009). Neuromyelitis optica: pathogenicity of patient immunoglobulin in vivo. Ann. Neurol..

[10] Linington C, Bradl M, Lassmann H, Brunner C, Vass K (1988). Augmentation of demyelination in rat acute allergic encephalomyelitis by circulating mouse monoclonal antibodies directed against a myelin/oligodendrocyte glycoprotein. Am. J. Pathol..

[11] Cross AH, Trotter JL, Lyons J-A (2001). B cells and antibodies in CNS demyelinating disease. J. Neuroimmunol..

[12] Carson MJ, Thrash JC, Walter B (2006). The cellular response in neuroinflammation: the role of leukocytes, microglia and astrocytes in neuronal death and survival. Clin. Neurosci. Res..

[13] Heneka MT (2015). Neuroinflammation in Alzheimer’s disease. Lancet Neurol..

[14] Hirsch EC, Hunot S (2009). Neuroinflammation in Parkinson’s disease: a target for neuroprotection?. Lancet Neurol..

[15] Vargas DL, Nascimbene C, Krishnan C, Zimmerman AW, Pardo CA (2005). Neuroglial activation and neuroinflammation in the brain of patients with autism. Ann. Neurol..

[16] Berer K (2017). Gut microbiota from multiple sclerosis patients enables spontaneous autoimmune encephalomyelitis in mice. Proc. Natl Acad. Sci. U. S. A..

[17] Mestre L (2019). Manipulation of gut microbiota influences immune responses, axon preservation, and motor disability in a model of progressive multiple sclerosis. Front. Immunol..

[18] Park J, Wang Q, Wu Q, Mao-Draayer Y, Kim CH (2019). Bidirectional regulatory potentials of short-chain fatty acids and their G-protein-coupled receptors in autoimmune neuroinflammation. Sci. Rep..

[19] Haase S (2020). The role of the gut microbiota and microbial metabolites in neuroinflammation. Eur. J. Immunol..

[20] Wong JM, De Souza R, Kendall CW, Emam A, Jenkins DJ (2006). Colonic health: fermentation and short chain fatty acids. J. Clin. Gastroenterol..

[21] Yano JM (2015). Indigenous bacteria from the gut microbiota regulate host serotonin biosynthesis. Cell.

[22] Alexeev EE (2018). Microbiota-derived indole metabolites promote human and murine intestinal homeostasis through regulation of interleukin-10 receptor. Am. J. Pathol..

[23] Taleb S (2019). Tryptophan dietary impacts gut barrier and metabolic diseases. Front. Immunol..

[24] Agus A, Planchais J, Sokol H (2018). Gut microbiota regulation of tryptophan metabolism in health and disease. Cell Host Microbe.

[25] Sayin SamaI (2013). Gut microbiota regulates bile acid metabolism by reducing the levels of tauro-beta-muricholic acid, a naturally occurring FXR Antagonist. Cell Metab..

[26] Kim CH (2018). Immune regulation by microbiome metabolites. Immunology.

[27] Luu M (2019). The short-chain fatty acid pentanoate suppresses autoimmunity by modulating the metabolic-epigenetic crosstalk in lymphocytes. Nat. Commun..

[28] Parker A, Fonseca S, Carding SR (2020). Gut microbes and metabolites as modulators of blood-brain barrier integrity and brain health. Gut Microbes.

[29] Macfarlane S, Macfarlane GT (2003). Regulation of short-chain fatty acid production. Proc. Nutr. Soc..

[30] Nøhr MK (2013). GPR41/FFAR3 and GPR43/FFAR2 as cosensors for short-chain fatty acids in enteroendocrine cells vs FFAR3 in enteric neurons and FFAR2 in enteric leukocytes. Endocrinology.

[31] Pluznick JL (2017). Microbial short-chain fatty acids and blood pressure regulation. Curr. Hypertens. Rep..

[32] Thangaraju M (2009). GPR109A is a G-protein-coupled receptor for the bacterial fermentation product butyrate and functions as a tumor suppressor in colon. Cancer Res..

[33] Sealy L, Chalkley R (1978). The effect of sodium butyrate on histone modification. Cell.

[34] Kobayashi M (2017). Short-chain fatty acids, GPR41 and GPR43 ligands, inhibit TNF-α-induced MCP-1 expression by modulating p38 and JNK signaling pathways in human renal cortical epithelial cells. Biochem. Biophys. Res. Commun..

[35] Yu X (2014). Short-chain fatty acids from periodontal pathogens suppress histone deacetylases, EZH2, and SUV39H1 to promote Kaposi’s sarcoma-associated herpesvirus replication. J. Virol..

[36] Kim CH, Park J, Kim M (2014). Gut microbiota-derived short-chain Fatty acids, T cells, and inflammation. Immune Netw..

[37] Säemann MD (2000). Anti‐inflammatory effects of sodium butyrate on human monocytes: potent inhibition of IL‐12 and up‐regulation of IL‐10 production. FASEB J..

[38] Sun M (2018). Microbiota-derived short-chain fatty acids promote Th1 cell IL-10 production to maintain intestinal homeostasis. Nat. Commun..

[39] Kim CH (2021). Control of lymphocyte functions by gut microbiota-derived short-chain fatty acids. Cell. Mol. Immunol..

[40] Sepahi A, Liu Q, Friesen L, Kim CH (2021). Dietary fiber metabolites regulate innate lymphoid cell responses. Mucosal Immunol..

[41] Chun E (2019). Metabolite-sensing receptor Ffar2 regulates colonic group 3 innate lymphoid cells and gut. Immun. Immun..

[42] Goverse G (2017). Diet-derived short chain fatty acids stimulate intestinal epithelial cells to induce mucosal tolerogenic dendritic cells. J. Immunol..

[43] Chen H (2019). A forward chemical genetic screen reveals gut microbiota metabolites that modulate host physiology. Cell.

[44] Proietti E, Rossini S, Grohmann U, Mondanelli G (2020). Polyamines and kynurenines at the intersection of immune modulation. Trends Immunol..

[45] Wlodarska M (2017). Indoleacrylic acid produced by commensal peptostreptococcus species suppresses inflammation. Cell Host Microbe.

[46] Lamas B (2016). CARD9 impacts colitis by altering gut microbiota metabolism of tryptophan into aryl hydrocarbon receptor ligands. Nat. Med..

[47] Zelante T (2013). Tryptophan catabolites from microbiota engage aryl hydrocarbon receptor and balance mucosal reactivity via interleukin-22. Immunity.

[48] Rouse M, Singh NP, Nagarkatti PS, Nagarkatti M (2013). Indoles mitigate the development of experimental autoimmune encephalomyelitis by induction of reciprocal differentiation of regulatory T cells and Th17 cells. Br. J. Pharmacol..

[49] Matsumoto M, Benno Y (2007). The relationship between microbiota and polyamine concentration in the human intestine: a pilot study. Microbiol. Immunol..

[50] Landau G, Bercovich Z, Park MH, Kahana C (2010). The role of polyamines in supporting growth of mammalian cells is mediated through their requirement for translation initiation and elongation. J. Biol. Chem..

[51] Tersey SA, Colvin SC, Maier B, Mirmira RG (2014). Protective effects of polyamine depletion in mouse models of type 1 diabetes: implications for therapy. Amino Acids.

[52] Chitrala KN, Yang X, Nagarkatti P, Nagarkatti M (2018). Comparative analysis of interactions between aryl hydrocarbon receptor ligand binding domain with its ligands: a computational study. BMC Struct. Biol..

[53] Hu Q (2013). Ginsenosides are novel naturally-occurring aryl hydrocarbon receptor ligands. PLoS One.

[54] Lee MJ (2016). Korean red ginseng and ginsenoside-Rb1/-Rg1 alleviate experimental autoimmune encephalomyelitis by suppressing Th1 and Th17 cells and upregulating regulatory T Cells. Mol. Neurobiol..

[55] Xuzhu G (2012). Resveratrol modulates murine collagen-induced arthritis by inhibiting Th17 and B-cell function. Ann. Rheum. Dis..

[56] Campbell C (2020). Bacterial metabolism of bile acids promotes generation of peripheral regulatory T cells. Nature.

[57] Hang S (2019). Bile acid metabolites control TH17 and Treg cell differentiation. Nature.

[58] Reddy BS, Watanabe K, Weisburger JH, Wynder EL (1977). Promoting effect of bile acids in colon carcinogenesis in germ-free and conventional F344 rats. Cancer Res.

[59] Wang YD, Chen WD, Yu D, Forman BM, Huang W (2011). The G-protein-coupled bile acid receptor, Gpbar1 (TGR5), negatively regulates hepatic inflammatory response through antagonizing nuclear factor kappa light-chain enhancer of activated B cells (NF-kappaB) in mice. Hepatology.

[60] Sakanaka T (2015). The effects of a TGR5 agonist and a dipeptidyl peptidase IV inhibitor on dextran sulfate sodium-induced colitis in mice. J. Gastroenterol. Hepatol..

[61] Hao H (2017). Farnesoid X receptor regulation of the NLRP3 inflammasome underlies cholestasis-associated sepsis. Cell Metab..

[62] Guo C (2016). Bile acids control inflammation and metabolic disorder through inhibition of NLRP3 inflammasome. Immunity.

[63] Wang Z (2011). Gut flora metabolism of phosphatidylcholine promotes cardiovascular disease. Nature.

[64] Yazici C (2017). Race-dependent association of sulfidogenic bacteria with colorectal cancer. Gut.

[65] Hsiao EY (2013). Microbiota modulate behavioral and physiological abnormalities associated with neurodevelopmental disorders. Cell.

[66] Guo Z (2016). Intestinal microbiota distinguish gout patients from healthy humans. Sci. Rep..

[67] Vogt NM (2018). The gut microbiota-derived metabolite trimethylamine N-oxide is elevated in Alzheimer’s disease. Alzheimers Res. Ther..

[68] Shao X (2016). Uric acid induces cognitive dysfunction through hippocampal inflammation in rodents and humans. J. Neurosci..

[69] Liu Y, Hou Y, Wang G, Zheng X, Hao H (2020). Gut microbial metabolites of aromatic amino acids as signals in host–microbe interplay. Trends Endocrinol. Metab..

[70] Veldhoen M (2008). The aryl hydrocarbon receptor links TH17-cell-mediated autoimmunity to environmental toxins. Nature.

[71] Sampson TR (2016). Gut microbiota regulate motor deficits and neuroinflammation in a model of Parkinson’s Disease. Cell.

[72] Sharon G (2019). Human gut microbiota from autism spectrum disorder promote behavioral symptoms in mice. Cell.

[73] Kaur H (2020). Effects of probiotic supplementation on short chain fatty acids in the AppNL-G-F mouse model of Alzheimer’s Disease. J. Alzheimers Dis..

[74] Haghikia A (2015). Dietary fatty acids directly impact central nervous system autoimmunity via the small intestine. Immunity.

[75] Constantinescu CS, Farooqi N, O’Brien K, Gran B (2011). Experimental autoimmune encephalomyelitis (EAE) as a model for multiple sclerosis (MS). Br. J. Pharmacol..

[76] Sun X, Liu Y, Liu B, Xiao Z, Zhang L (2012). Rolipram promotes remyelination possibly via MEK-ERK signal pathway in cuprizone-induced demyelination mouse. Exp. Neurol..

[77] Blakemore WF (1973). Demyelination of the superior cerebellar peduncle in the mouse induced by cuprizone. J. Neurol. Sci..

[78] Pavelko KD, Van Engelen BG, Rodriguez M (1998). Acceleration in the rate of CNS remyelination in lysolecithin-induced demyelination. J. Neurosci..

[79] Lipton HL (1975). Theiler’s virus infection in mice: an unusual biphasic disease process leading to demyelination. Infect. Immun..

[80] Liu C, Collins J, Sharp E (1967). The pathogenesis of Theiler’s GD VII encephalomyelitis virus infection in mice as studied by immunofluorescent technique and infectivity titrations. J. Immunol..

[81] Krishnamoorthy G, Lassmann H, Wekerle H, Holz A (2006). Spontaneous opticospinal encephalomyelitis in a double-transgenic mouse model of autoimmune T cell/B cell cooperation. J. Clin. Invest..

[82] Pöllinger B (2009). Spontaneous relapsing-remitting EAE in the SJL/J mouse: MOG-reactive transgenic T cells recruit endogenous MOG-specific B cells. J. Exp. Med..

[83] Johanson DM (2020). Experimental autoimmune encephalomyelitis is associated with changes of the microbiota composition in the gastrointestinal tract. Sci. Rep..

[84] Berer K (2018). Dietary non-fermentable fiber prevents autoimmune neurological disease by changing gut metabolic and immune status. Sci. Rep..

[85] Lee YK, Menezes JS, Umesaki Y, Mazmanian SK (2011). Proinflammatory T-cell responses to gut microbiota promote experimental autoimmune encephalomyelitis. Proc. Natl Acad. Sci. U. S. A..

[86] Ochoa-Reparaz J (2009). Role of gut commensal microflora in the development of experimental autoimmune encephalomyelitis. J. Immunol..

[87] Benedek G (2017). Estrogen protection against EAE modulates the microbiota and mucosal-associated regulatory cells. J. Neuroimmunol..

[88] Cekanaviciute E (2017). Gut bacteria from multiple sclerosis patients modulate human T cells and exacerbate symptoms in mouse models. Proc. Natl Acad. Sci. U. S. A..

[89] Visser L (2005). Proinflammatory bacterial peptidoglycan as a cofactor for the development of central nervous system autoimmune disease. J. Immunol..

[90] Ivanov II (2008). Specific microbiota direct the differentiation of IL-17-producing T-helper cells in the mucosa of the small intestine. Cell Host Microbe.

[91] Calcinotto A (2018). Microbiota-driven interleukin-17-producing cells and eosinophils synergize to accelerate multiple myeloma progression. Nat. Commun..

[92] Chen H (2019). Gut microbiota interventions with clostridium butyricum and norfloxacin modulate immune response in experimental autoimmune encephalomyelitis mice. Front. Immunol..

[93] Ramakrishna C (2019). Bacteroides fragilis polysaccharide A induces IL-10 secreting B and T cells that prevent viral encephalitis. Nat. Commun..

[94] Ochoa-Reparaz J (2010). A polysaccharide from the human commensal Bacteroides fragilis protects against CNS demyelinating disease. Mucosal Immunol..

[95] Lee K (2012). Bacillus-derived poly-gamma-glutamic acid reciprocally regulates the differentiation of T helper 17 and regulatory T cells and attenuates experimental autoimmune encephalomyelitis. Clin. Exp. Immunol..

[96] Felts PA (2005). Inflammation and primary demyelination induced by the intraspinal injection of lipopolysaccharide. Brain.

[97] Minagar A, Alexander JS (2003). Blood-brain barrier disruption in multiple sclerosis. Mult. Scler..

[98] Dopkins N, Nagarkatti PS, Nagarkatti M (2018). The role of gut microbiome and associated metabolome in the regulation of neuroinflammation in multiple sclerosis and its implications in attenuating chronic inflammation in other inflammatory and autoimmune disorders. Immunology.

[99] Braniste V (2014). The gut microbiota influences blood-brain barrier permeability in mice. Sci. Transl. Med..

[100] Erny D (2015). Host microbiota constantly control maturation and function of microglia in the CNS. Nat. Neurosci..

[101] Chen T, Noto D, Hoshino Y, Mizuno M, Miyake S (2019). Butyrate suppresses demyelination and enhances remyelination. J. Neuroinflamm..

[102] Mikov M, Kevresan S, Kuhajda K, Jakovljevic V, Vasovic V (2004). 3Alpha,7alpha-dihydroxy-12-oxo-5beta-cholanate as blood-brain barrier permeator. Pol. J. Pharmacol..

[103] Greenwood J, Adu J, Davey AJ, Abbott NJ, Bradbury MW (1991). The effect of bile salts on the permeability and ultrastructure of the perfused, energy-depleted, rat blood-brain barrier. J. Cereb. Blood Flow. Metab..

[104] Haghikia A (2016). Dietary fatty acids directly impact central nervous system autoimmunity via the small intestine. Immunity.

[105] Mizuno M, Noto D, Kaga N, Chiba A, Miyake S (2017). The dual role of short fatty acid chains in the pathogenesis of autoimmune disease models. PLoS One.

[106] Schneeman BO, Gallaher D (1980). Changes in small intestinal digestive enzyme activity and bile acids with dietary cellulose in rats. J. Nutr..

[107] Levrat M-A, Behr SR, Rémésy C, Demigné C (1991). Effects of soybean fiber on cecal digestion in rats previously adapted to a fiber-free diet. J. Nutr..

[108] Fischer F (2020). Dietary cellulose induces anti-inflammatory immunity and transcriptional programs via maturation of the intestinal microbiota. Gut Microbes.

[109] Park J (2015). Short-chain fatty acids induce both effector and regulatory T cells by suppression of histone deacetylases and regulation of the mTOR–S6K pathway. Mucosal Immunol..

[110] Cox MA (2009). Short-chain fatty acids act as antiinflammatory mediators by regulating prostaglandin E(2) and cytokines. World J. Gastrol..

[111] Smith PM (2013). The microbial metabolites, short-chain fatty acids, regulate colonic Treg cell homeostasis. Science.

[112] Kim MH, Kang SG, Park JH, Yanagisawa M, Kim CH (2013). Short-chain fatty acids activate GPR41 and GPR43 on intestinal epithelial cells to promote inflammatory responses in mice. Gastroenterology.

[113] Macia L (2015). Metabolite-sensing receptors GPR43 and GPR109A facilitate dietary fibre-induced gut homeostasis through regulation of the inflammasome. Nat. Commun..

[114] Quintana FJ (2008). Control of T(reg) and T(H)17 cell differentiation by the aryl hydrocarbon receptor. Nature.

[115] Quintana FJ (2010). An endogenous aryl hydrocarbon receptor ligand acts on dendritic cells and T cells to suppress experimental autoimmune encephalomyelitis. Proc. Natl Acad. Sci. U. S. A..

[116] Rothhammer V (2016). Type I interferons and microbial metabolites of tryptophan modulate astrocyte activity and central nervous system inflammation via the aryl hydrocarbon receptor. Nat. Med..

[117] Giacoppo S (2013). Protective role of (RS)-glucoraphanin bioactivated with myrosinase in an experimental model of multiple sclerosis. CNS Neurosci. Ther..

[118] Saresella M (2020). Alterations in circulating fatty acid are associated with gut microbiota dysbiosis and inflammation in multiple sclerosis. Front. Immunol..

[119] Correale J, Gaitan MI, Ysrraelit MC, Fiol MP (2017). Progressive multiple sclerosis: from pathogenic mechanisms to treatment. Brain.

[120] Branton WG (2016). Brain microbiota disruption within inflammatory demyelinating lesions in multiple sclerosis. Sci. Rep..

[121] Miyake S (2015). Dysbiosis in the gut microbiota of patients with multiple sclerosis, with a striking depletion of species belonging to Clostridia XIVa and IV Clusters. PLoS One.

[122] Zeng Q (2019). Gut dysbiosis and lack of short chain fatty acids in a Chinese cohort of patients with multiple sclerosis. Neurochem. Int..

[123] Schrijver IA (2001). Bacterial peptidoglycan and immune reactivity in the central nervous system in multiple sclerosis. Brain.

[124] Campbell DJ, Butcher EC (2002). Intestinal attraction: CCL25 functions in effector lymphocyte recruitment to the small intestine. J. Clin. Invest..

[125] Kang SG, Wang C, Matsumoto S, Kim CH (2009). High and low vitamin A therapies induce distinct FoxP3+ T-cell subsets and effectively control intestinal inflammation. Gastroenterology.

[126] Liu C (2006). Coordination between CCR7- and CCR9-mediated chemokine signals in prevascular fetal thymus colonization. Blood.

[127] Uehara S, Song K, Farber JM, Love PE (2002). Characterization of CCR9 expression and CCL25/thymus-expressed chemokine responsiveness during T cell development: CD3(high)CD69+ thymocytes and gammadeltaTCR+ thymocytes preferentially respond to CCL25. J. Immunol..

[128] Wang C, Kang SG, Lee J, Sun Z, Kim CH (2009). The roles of CCR6 in migration of Th17 cells and regulation of effector T-cell balance in the gut. Mucosal Immunol..

[129] Kadowaki A, Saga R, Lin Y, Sato W, Yamamura T (2019). Gut microbiota-dependent CCR9+CD4+ T cells are altered in secondary progressive multiple sclerosis. Brain.

[130] Miller PG, Bonn MB, Franklin CL, Ericsson AC, McKarns SC (2015). TNFR2 deficiency acts in concert with gut microbiota to precipitate spontaneous sex-biased central nervous system demyelinating autoimmune disease. J. Immunol..

[131] Saresella M (2017). Immunological and clinical effect of diet modulation of the gut microbiome in multiple sclerosis patients: a pilot study. Front. Immunol..

[132] Takewaki D (2020). Alterations of the gut ecological and functional microenvironment in different stages of multiple sclerosis. Proc. Natl Acad. Sci. U. S. A..

[133] Chen J (2016). Multiple sclerosis patients have a distinct gut microbiota compared to healthy controls. Sci. Rep..

[134] Duscha A (2020). Propionic acid shapes the multiple sclerosis disease course by an immunomodulatory mechanism. Cell.

[135] Perez-Perez S (2020). Acetate correlates with disability and immune response in multiple sclerosis. PeerJ.

[136] Moussallieh FM (2014). Serum analysis by 1H nuclear magnetic resonance spectroscopy: a new tool for distinguishing neuromyelitis optica from multiple sclerosis. Mult. Scler..

[137] Crabtree B, Souter MJ, Anderson SE (1989). Evidence that the production of acetate in rat hepatocytes is a predominantly cytoplasmic process. Biochem. J..

[138] Hyyppä MT, Jolma T, Riekkinen P, Rinne UK (1975). Effects of L-tryptophan treatment on central indoleamine metabolism and short-lasting neurologic disturbances in multiple sclerosis. J. Neural Transm..

[139] Hyyppä MT, Jolma T, Långvik V-A, Kytömäki O, Syvälahti E (1977). l-Tryptophan and neuroendocrine regulation in neurologic patients: Hormone responses to l-tryptophan loading in patients with hypothalamic lesions. Psychoneuroendocrinology.

[140] Gaetani L (2020). Host and microbial tryptophan metabolic profiling in multiple sclerosis. Front . Immunol..

[141] Lim CK (2017). Kynurenine pathway metabolomics predicts and provides mechanistic insight into multiple sclerosis progression. Sci. Rep..

[142] Nourbakhsh B (2018). Altered tryptophan metabolism is associated with pediatric multiple sclerosis risk and course. Ann. Clin. Transl. Neurol..

[143] Bhargava P (2020). Bile acid metabolism is altered in multiple sclerosis and supplementation ameliorates neuroinflammation. J. Clin. Invest..

[144] Lieben CK (2018). Intake of tryptophan-enriched whey protein acutely enhances recall of positive loaded words in patients with multiple sclerosis. Clin. Nutr..

[145] Gorman MP, Healy BC, Polgar-Turcsanyi M, Chitnis T (2009). Increased relapse rate in pediatric-onset compared with adult-onset multiple sclerosis. Arch. Neurol..

[146] Tremlett H, Waubant E (2018). The gut microbiota and pediatric multiple sclerosis: recent findings. Neurotherapeutics.

[147] Heath-Pagliuso S (1998). Activation of the Ah receptor by tryptophan and tryptophan metabolites. Biochemistry.

[148] Opitz CA (2011). An endogenous tumour-promoting ligand of the human aryl hydrocarbon receptor. Nature.

[149] Wang J (2006). Kynurenic acid as a ligand for orphan G protein-coupled receptor GPR35. J. Biol. Chem..

[150] Rothhammer V (2018). Microglial control of astrocytes in response to microbial metabolites. Nature.

[151] Maetzler W, Berg D (2018). Parkinson disease in 2017: changing views after 200 years of Parkinson disease. Nat. Rev. Neurol..

[152] Singh M (2008). Polymorphism in environment responsive genes and association with Parkinson disease. Mol. Cell. Biochem..

[153] Spillantini MG (1997). Alpha-synuclein in Lewy bodies. Nature.

[154] Winner B (2011). In vivo demonstration that alpha-synuclein oligomers are toxic. Proc. Natl Acad. Sci. U. S. A..

[155] Kustrimovic N (2018). Parkinson’s disease patients have a complex phenotypic and functional Th1 bias: cross-sectional studies of CD4+ Th1/Th2/T17 and Treg in drug-naive and drug-treated patients. J. Neuroinflamm..

[156] Chen S (2017). Increased abundance of myeloid-derived suppressor cells and Th17 cells in peripheral blood of newly-diagnosed Parkinson’s disease patients. Neurosci. Lett..

[157] Hernandez DG, Reed X, Singleton AB (2016). Genetics in Parkinson disease: Mendelian versus non-Mendelian inheritance. J. Neurochem..

[158] Fujioka S (2017). Occurrence of Crohn’s disease with Parkinson’s disease. Parkinsonism Relat. Disord..

[159] Franke A (2010). Genome-wide meta-analysis increases to 71 the number of confirmed Crohn’s disease susceptibility loci. Nat. Genet..

[160] Unger MM (2016). Short chain fatty acids and gut microbiota differ between patients with Parkinson’s disease and age-matched controls. Parkinsonism Relat. Disord..

[161] Keshavarzian A (2015). Colonic bacterial composition in Parkinson’s disease. Mov. Disord..

[162] Perez-Pardo P (2019). Role of TLR4 in the gut-brain axis in Parkinson’s disease: a translational study from men to mice. Gut.

[163] Hou YF (2021). Gut microbiota-derived propionate mediates the neuroprotective effect of osteocalcin in a mouse model of Parkinson’s disease. Microbiome.

[164] Bordoni L (2019). Positive effect of an electrolyzed reduced water on gut permeability, fecal microbiota and liver in an animal model of Parkinson’s disease. PLoS One.

[165] Li P (2021). Gut microbiota dysbiosis is associated with elevated bile acids in Parkinson’s Disease. Metabolites.

[166] Shao Y (2021). Comprehensive metabolic profiling of Parkinson’s disease by liquid chromatography-mass spectrometry. Mol. Neurodegener..

[167] Cirstea MS (2020). Microbiota composition and metabolism are associated with gut function in Parkinson’s Disease. Mov. Disord..

[168] Funayama M (2005). An LRRK2 mutation as a cause for the parkinsonism in the original PARK8 family. Ann. Neurol..

[169] Park J (2017). Parkinson disease-associated LRRK2 G2019S transgene disrupts marrow myelopoiesis and peripheral Th17 response. J. Leukoc. Biol..

[170] Ekstrand MI (2007). Progressive parkinsonism in mice with respiratory-chain-deficient dopamine neurons. Proc. Natl Acad. Sci. U. S. A..

[171] Liu J (2017). Sodium butyrate exerts protective effect against Parkinson’s disease in mice via stimulation of glucagon like peptide-1. J. Neurol. Sci..

[172] Johnson J (2018). Valproate and sodium butyrate attenuate manganese-decreased locomotor activity and astrocytic glutamate transporters expression in mice. Neurotoxicology.

[173] Kidd SK, Schneider JS (2011). Protective effects of valproic acid on the nigrostriatal dopamine system in a 1-methyl-4-phenyl-1,2,3,6-tetrahydropyridine mouse model of Parkinson’s disease. Neuroscience.

[174] Aho VTE (2021). Relationships of gut microbiota, short-chain fatty acids, inflammation, and the gut barrier in Parkinson’s disease. Mol. Neurodegener..

[175] Qiao CM (2020). Sodium butyrate exacerbates Parkinson’s disease by aggravating neuroinflammation and colonic inflammation in MPTP-Induced Mice Model. Neurochem. Res..

[176] Park J, Goergen CJ, HogenEsch H, Kim CH (2016). Chronically elevated levels of short-chain fatty acids induce T cell-mediated ureteritis and hydronephrosis. J. Immunol..

[177] Graham SF (2018). Metabolomic profiling of bile acids in an experimental model of prodromal Parkinson’s Disease. Metabolites.

[178] Cuevas, E. et al. Tauroursodeoxycholic acid (TUDCA) is neuroprotective in a chronic mouse model of Parkinson’s disease. *Nutr. Neurosci*. 1–18 (2020). 10.1080/1028415X.2020.1859729 33345721

[179] Castro-Caldas M (2012). Tauroursodeoxycholic acid prevents MPTP-induced dopaminergic cell death in a mouse model of Parkinson’s disease. Mol. Neurobiol..

[180] Duan WM, Rodrigues CM, Zhao LR, Steer CJ, Low WC (2002). Tauroursodeoxycholic acid improves the survival and function of nigral transplants in a rat model of Parkinson’s disease. Cell Transpl..

[181] Rosa AI (2018). Tauroursodeoxycholic acid improves motor symptoms in a mouse model of Parkinson’s Disease. Mol. Neurobiol..

[182] Moreira S (2017). Nrf2 activation by tauroursodeoxycholic acid in experimental models of Parkinson’s disease. Exp. Neurol..

[183] Mortiboys H (2015). UDCA exerts beneficial effect on mitochondrial dysfunction in LRRK2(G2019S) carriers and in vivo. Neurology.

[184] Tiwari S, Atluri V, Kaushik A, Yndart A, Nair M (2019). Alzheimer’s disease: pathogenesis, diagnostics, and therapeutics. Int. J. Nanomed..

[185] Fujii Y (2019). Fecal metabolite of a gnotobiotic mouse transplanted with gut microbiota from a patient with Alzheimer’s disease. Biosci. Biotechnol. Biochem..

[186] Nagpal R (2020). Gut mycobiome and its interaction with diet, gut bacteria and alzheimer’s disease markers in subjects with mild cognitive impairment: a pilot study. EBioMedicine.

[187] Marizzoni M (2020). Short-chain fatty acids and lipopolysaccharide as mediators between gut dysbiosis and amyloid pathology in Alzheimer’s Disease. J. Alzheimers Dis..

[188] Ho L (2018). Protective roles of intestinal microbiota derived short chain fatty acids in Alzheimer’s disease-type beta-amyloid neuropathological mechanisms. Expert. Rev. Neurother..

[189] Liu S, Gao J, Zhu M, Liu K, Zhang HL (2020). Gut microbiota and dysbiosis in Alzheimer’s Disease: implications for pathogenesis and treatment. Mol. Neurobiol..

[190] Luchsinger JA, Tang M-X, Shea S, Mayeux R (2002). Caloric intake and the risk of Alzheimer Disease. Arch. Neurol..

[191] Engelhart MJ (2002). Dietary intake of antioxidants and risk of Alzheimer disease. JAMA.

[192] Spychala MS (2018). Age-related changes in the gut microbiota influence systemic inflammation and stroke outcome. Ann. Neurol..

[193] Govindarajan N, Agis-Balboa RC, Walter J, Sananbenesi F, Fischer A (2011). Sodium butyrate improves memory function in an Alzheimer’s disease mouse model when administered at an advanced stage of disease progression. J. Alzheimers Dis..

[194] Martins IJ, Fernando WMADB (2014). High fibre diets and Alzheimer’s Disease. Food Nutr. Sci..

[195] Sun J (2020). Effect of Clostridium butyricum against microglia-mediated neuroinflammation in Alzheimer’s Disease via regulating gut microbiota and metabolites Butyrate. Mol. Nutr. Food Res..

[196] Da Silva SV (2016). Early synaptic deficits in the APP/PS1 mouse model of Alzheimer’s disease involve neuronal adenosine A 2A receptors. Nat. Commun..

[197] Spires TL, Hyman BT (2005). Transgenic models of Alzheimer’s disease: learning from animals. NeuroRx.

[198] Trinchese F (2004). Progressive age-related development of Alzheimer-like pathology in APP/PS1 mice. Ann. Neurol..

[199] Colombo AV (2021). Microbiota-derived short chain fatty acids modulate microglia and promote Aβ plaque deposition. eLife.

[200] Sun J (2019). Fecal microbiota transplantation alleviated Alzheimer’s disease-like pathogenesis in APP/PS1 transgenic mice. Transl. Psychiatr..

[201] Widner B (2000). Tryptophan degradation and immune activation in Alzheimer’s disease. J. Neural Transm. (Vienna).

[202] MahmoudianDehkordi S (2019). Altered bile acid profile associates with cognitive impairment in Alzheimer’s disease-An emerging role for gut microbiome. Alzheimers Dement.

[203] Baloni P (2020). Metabolic network analysis reveals altered bile acid synthesis and metabolism in Alzheimer’s Disease. Cell Rep. Med.

[204] Lord C, Elsabbagh M, Baird G, Veenstra-Vanderweele J (2018). Autism spectrum disorder. Lancet.

[205] Bölte S, Girdler S, Marschik PB (2019). The contribution of environmental exposure to the etiology of autism spectrum disorder. Cell. Mol. Life Sci..

[206] Choi GB (2016). The maternal interleukin-17a pathway in mice promotes autism-like phenotypes in offspring. Science.

[207] Chaidez V, Hansen RL, Hertz-Picciotto I (2014). Gastrointestinal problems in children with autism, developmental delays or typical development. J. Autism Dev. Disord..

[208] Madra M, Ringel R, Margolis KG (2020). Gastrointestinal issues and autism spectrum disorder. Child Adolesc. Psychiatr. N. Am..

[209] De Angelis M (2013). Fecal microbiota and metabolome of children with autism and pervasive developmental disorder not otherwise specified. PLoS One.

[210] Kim S (2017). Maternal gut bacteria promote neurodevelopmental abnormalities in mouse offspring. Nature.

[211] Adams JB, Johansen LJ, Powell LD, Quig D, Rubin RA (2011). Gastrointestinal flora and gastrointestinal status in children with autism – comparisons to typical children and correlation with autism severity. BMC Gastroenterol..

[212] Liu S (2019). Altered gut microbiota and short chain fatty acids in Chinese children with autism spectrum disorder. Sci. Rep..

[213] Choi J (2018). Pathophysiological and neurobehavioral characteristics of a propionic acid-mediated autism-like rat model. PLoS One.

[214] Abdelli LS, Samsam A, Naser SA (2019). Propionic acid induces gliosis and neuro-inflammation through modulation of PTEN/AKT pathway in Autism Spectrum Disorder. Sci. Rep..

[215] Kratsman N, Getselter D, Elliott E (2016). Sodium butyrate attenuates social behavior deficits and modifies the transcription of inhibitory/excitatory genes in the frontal cortex of an autism model. Neuropharmacology.

[216] Hammer A (2017). Impact of combined sodium chloride and saturated long-chain fatty acid challenge on the differentiation of T helper cells in neuroinflammation. J. Neuroinflamm..

[217] Platten M (2005). Treatment of autoimmune neuroinflammation with a synthetic tryptophan metabolite. Science.

[218] Sun MF (2018). Neuroprotective effects of fecal microbiota transplantation on MPTP-induced Parkinson’s disease mice: Gut microbiota, glial reaction and TLR4/TNF-alpha signaling pathway. Brain Behav. Immun..

[219] Shin C, Lim Y, Lim H, Ahn TB (2020). Plasma short-chain fatty acids in patients With Parkinson’s Disease. Mov. Disord..

[220] Chen SJ (2020). The gut metabolite trimethylamine N-oxide is associated with Parkinson’s Disease severity and progression. Mov. Disord..

[221] Coretti L (2018). Gut microbiota features in young children with Autism Spectrum Disorders. Front. Microbiol..

[222] Wang L (2012). Elevated fecal short chain fatty acid and ammonia concentrations in children with Autism Spectrum Disorder. Dig. Dis. Sci..

